# Adenovirus expressing dual c-Met-specific shRNA exhibits potent antitumor effect through autophagic cell death accompanied by senescence-like phenotypes in glioblastoma cells

**DOI:** 10.18632/oncotarget.3018

**Published:** 2015-02-17

**Authors:** Jung-Sun Lee, Eonju Oh, Ji Young Yoo, Kyeong Sook Choi, Mi Jin Yoon, Chae-Ok Yun

**Affiliations:** ^1^ Institute for Cancer Research, Yonsei University College of Medicine, Seoul, Korea; ^2^ Department of Molecular Science & Technology, Institute for Medical Sciences, Ajou University School of Medicine, Suwon, Korea; ^3^ Department of Bioengineering, College of Engineering, Hanyang University, Seoul, Korea

**Keywords:** adenovirus, autophagic cell death, cancer gene therapy, c-Met, short hairpin RNA (shRNA)

## Abstract

c-Met, a cognate receptor tyrosine kinase of hepatocyte growth factor, is overexpressed and/or mutated in number of tumors. Therefore, abrogation of c-Met signaling may serve as potential therapeutic targets. In this study, we generated Ads expressing single shRNA specific to c-Met (shMet) (dl/shMet4 and dl/shMet5) or dual shRNAs specific to c-Met (dl/shMet4+5); and examined the therapeutic potential of these newly engineered Ads in targeting c-Met, and delineated their mechanism of action *in vitro* and *in vivo*. Ads expressing shMet induced knock-down in c-Met, and phenotypically resulted in autophagy-like features including appearance of membranousvacuoles, formation of acidic vesicular organelles, and cleavage and recruitment of microtubule-associated protein1 light chain 3 to autophagosomes. Ads expressing shMet also suppressed Akt phosphorylation and increased number of senescence-related gene products including SM22, TGase II, and PAI-1. These changes resulted in inhibition of cell proliferation and G_2_/M arrest of U343 cells. *In vivo*, intratumoral injection with dl/shMet4+5 resulted in a significant reduction of tumor growth with corresponding increasing overall survival. Histopathological analysis of these treated tumors revealed that Atg5 was highly up-regulated, indicating the therapeutic induction of autophagy. In sum, these results reveal that autophagic cell death induced by shMet-expressing Ads provide a novel strategy for targeting c-Met-expressing tumors through non-apoptotic mechanism of cell death.

## INTRODUCTION

Receptor tyrosine kinases (RTKs) regulate many key processes in mammalian cell growth and survival, organ morphogenesis, neovascularization, and tissue repair and regeneration. Over-expression, activating mutations and/or defective downregulation of RTKs has been implicated as causative factors in the development and progression of numerous human cancers. To date over 90 known human RTKs have been characterized [1], and many are known to be proto-oncogenes driving tumorigenesis [2]; with c-Kit, Ephs, PDGF, Flt3, and c-Met being exemplars of this class [3].

c-Met is a high-affinity cognate receptor of its natural ligand hepatocyte growth factor (HGF)/scatter factor. HGF is expressed and released from surrounding stromal and mesenchymal cells and acts in a paracrine manner on c-Met-expressing tumor cells. Upon binding, c-Met undergoes autophosphorylation of specific tyrosine residues (Y1230/1234/1235), and activates its intrinsic kinase activity. c-Met alterations, including overexpression and/or activating mutations, have been described in a myriad of cancers [4, 5]; with most common mutations occurring at the cytoplasmic activation loop domain. Another common mutation occurs at the juxtamembrane domain, which plays a critical role in regulation of its catalytic function. These activating mutations then result in driving the activity and progression of c-Met driven tumors.

Due to its importance as an oncogenic driver, c-Met represents an attractive therapeutically relevant target of pharmacologic intervention. The most advanced c-Met inhibitor is Pfizer Inc.'s Xalkori crizotinib, a dual inhibitor of c-Met receptor tyrosine kinase and anaplastic lymphoma kinase (ALK) and their oncogenic variants. The drug is approved for advanced or metastatic non-small cell lung cancer (NSCLC) in patients whose tumors are ALK-positive. Behind Xalkori are tivantinib (ARQ 197) from ArQule Inc. and cabozantinib (XL184) from Exelixis Inc. The molecules are in Phase III testing for NSCLC and thyroid cancer, respectively [6, 7]. In addition, HGF neutralizing monoclonal antibodies [8], c-*met* antisense oligonucleotides [9], dominant-negative forms of the c-Met protein [10], ribozyme-mediated reduction of c-Met expression [11, 12], and dual-function decoy c-Met receptor [13] are also being investigated as possible strategies to block c-Met activation and suppress tumor growth, invasion, and metastasis.

Recently, RNA interference (RNAi) has also emerged as a new modality to silence gene expression. RNAi is a highly sequence-specific, gene-silencing process that works through double stranded RNA molecules that are homologous to the sequence of the target gene [14, 15]. DNA vector-mediated RNAi technology has made it possible to develop therapeutically applicable use of this technology in mammalian cells. Several examples using retroviral or adenoviral (Ad) vector systems to deliver siRNA for stable or transient expression, respectively, have been reported [16–18].

In this study, we show for the first time that inhibition of c-Met by Ad-mediated shRNA (dl/shMet4, dl/shMet5, and dl/shMet4+5) expression results in robust anti-tumor efficacy via autophagic cell death in various cancer cells. In addition, we observed that reduced c-Met expression induces dramatic inhibition of cancer cell proliferation by a senescence mechanism. We further found that dl/shMet4+5 mediates autophagic cell death, as indicated by accumulation LC3-II protein and autophagic vacuoles. Furthermore, the growth of established U343 human glioma xenograft was significantly suppressed by dl/shMet4+5. These observations strongly suggest that inhibition of c-Met via dual c-Met specific shRNA-expressing Ad is a viable approach to the treatment of c-Met driven tumor types and warrants further testing in the clinic.

## RESULTS

### Generation of recombinant Ads expressing shRNA specific to c-Met

**Figure 1:**
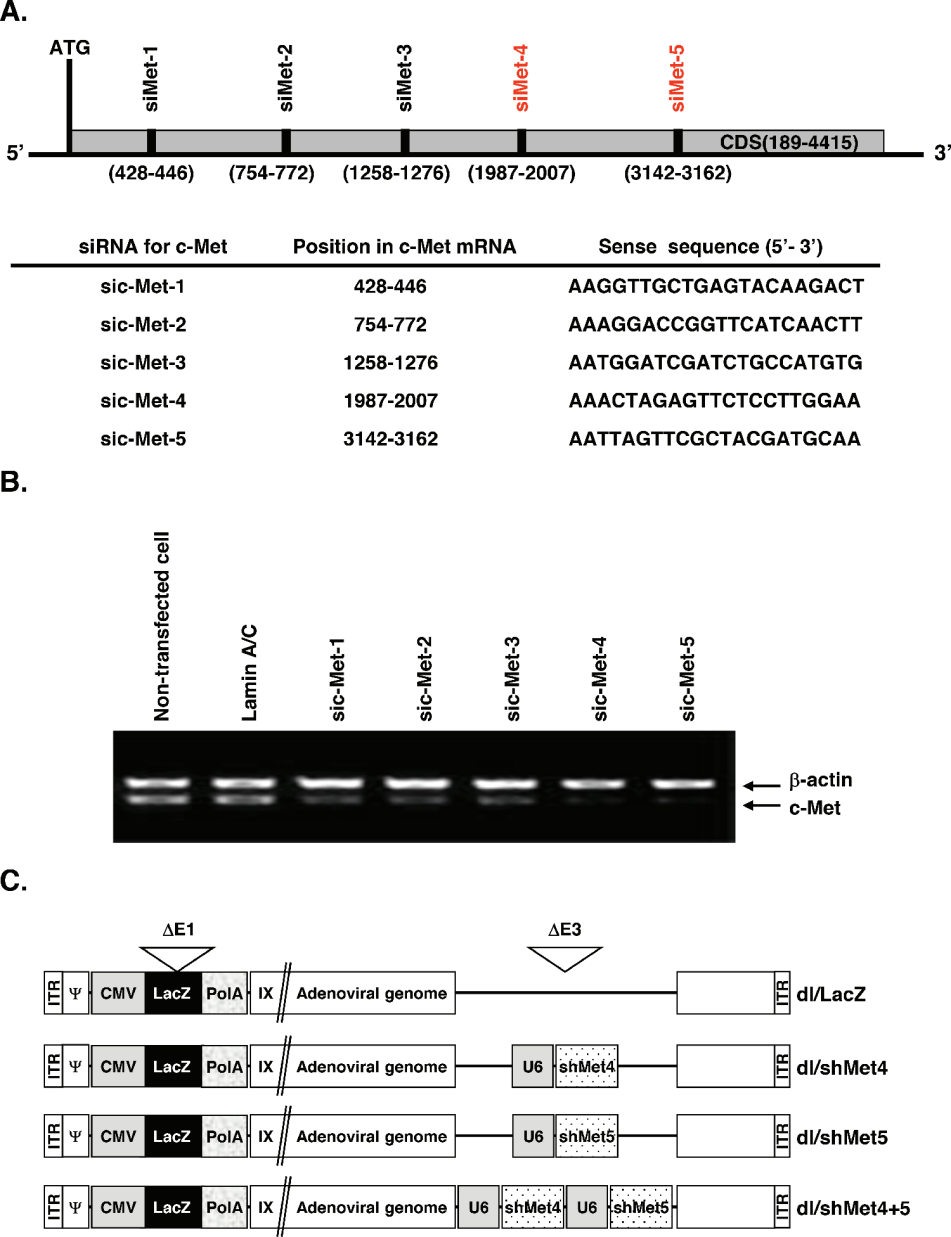
Schematic and characterization of c-Met RNAi target site **(A)** Location of five c-Met-specific siRNAs examined in this study. The target sequences within c-Met are shown. **(B)** shRNA-mediated *in vitro* knockdown of c-Met gene. Cells were transfected for 48 hr with pSP72/U6-sic-Met1, pSP72/U6-sic-Met2, pSP72/U6-sic-Met3, pSP72/U6-sic-Met4, or pSP72/U6-sic-Met5. LaminA/C was used as negative control. The knockdown of endogenous expression was measured by reverse transcriptase-polymerase chain reaction (RT-PCR) for c-Met. The experiment was repeated three times with reproducible results. **(C)** Schematic representation of the genomic structures of dl/LacZ, dl/shMet4, dl/shMet5, and dl/shMet4+5 adenoviruses used in this study.

To identify potent and effective siRNA targeting c-Met, siRNAs sequences spanning the cytoplasmic domain of c-Met (gi:4557746) were generated and examined in high c-Met-expressing U343 human glioma cell line (Figure 1A). To monitor potential off-target effects, lamin A/C-specific siRNA was used as a negative control. From this initial set, we identified two siRNAs (c-Met-4 and c-Met-5) that potently suppressed endogenous expression of c-Met mRNA (> 90%) (Figure 1B). As expected, lamin A/C-specific siRNA resulted in no significant alteration of c-Met RNA expression in comparison to non-transfected cells. Finally, as shown on Figure 1C, recombinant Ads expressing single c-Met shRNA No. 4 or No. 5 (dl/shMet4 or dl/shMet5) and expressing dual shRNA for c-Met (dl/shMet4+5) under the control of the human U6 promoter were generated.

### Suppression of c-Met expression by Ads expressing shMet4, shMet5, or shMet4+5

**Figure 2:**
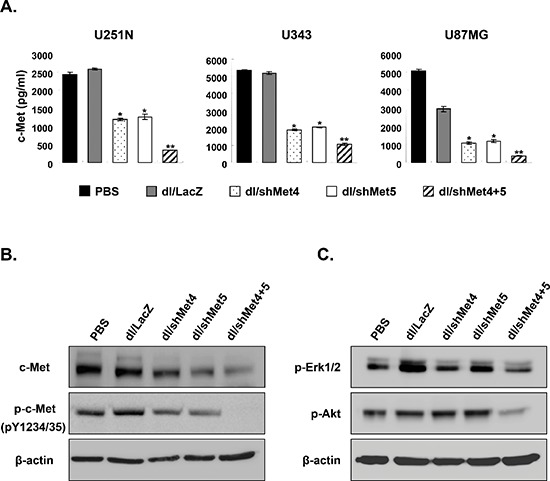
The expression of total c-Met, phospho-c-Met, phospho-Erk, and phospho-Akt in cancer cells transduced with c-Met-specific shRNA-expressing Ad **(A)** Various human glioma cancer cell lines were transduced with dl/LacZ, dl/shMet4, dl/shMet5, or dl/shMet4+5 (200 MOI for U251N and U87MG, 100 MOI for U343). c-Met concentration in the culture supernatant was measured at 72 hr after transduction by c-Met ELISA. Data are expressed as the mean ± SE (n = 3), and are representative of at least three independent experiments. **(B, C)** U343 glioma cells were transduced with dl/LacZ, dl/shMet4, dl/shMet5, or dl/shMet4+5 at 100 MOI. After 3 days, cells were harvested, and the expression levels of total c-Met, phospho-c-Met, phospho-Erk, and phospho-Akt were observed by western blot analysis. Twenty micrograms of protein was loaded in each lane.

To assess the efficiency of these newly engineered Ads to suppress c-Met, multiple human glioma cell lines (U251N, U343, and U87MG) and human normal fibroblast cell line (HDF) were transduced with dl/LacZ, dl/shMet4, dl/shMet5, or dl/shMet4+5. Following 3 days post-transduction, conditioned media from transduced cells was harvested and assayed to determine the amounts of c-Met protein. As shown in Figure 2A as expected, c-Met expression was dramatically suppressed by all three Ads, with the dual shRNA-expressing Ad showing the greatest knock-down. More specifically, after transduction with dl/shMet4+5, c-Met levels were significantly reduced by 86.9% (*P* < 0.01) compared to control Ad (dl/LacZ)-transduced in U251N cells, whereas the reduction was 53.9% and 51.1% with dl/shMet4 or dl/shMet5, respectively (*P* < 0.05). This enhanced efficiency of c-Met knockdown by dl/shMet4+5 was also observed in U343 (87.6%) and U87MG (91.9%) cells compared with dl/LacZ controls (*P* < 0.01). The expression levels of both phospho-c-Met and total c-Met were also markedly decreased in the dl/shMet4+5-transduced U343 compared with PBS-, dl/LacZ-, dl/shMet4-, or dl/shMet5-transduced cells (Figure 2B). In addition, phospholylated AKT (survival) and mitogen-activated protein kinase ERK1/2 (proliferation–differentiation) were both abrogated in the U343 cells treated with dl/shMet4+5 (Figure 2C). Similar results were observed in U251N and U87MG transduced with shMet-expressing Ads, showing the repressed total c-Met and phospho-Erk1/2 (Supplementary Figure S1). However, the expression of phospho-c-Met and phospho-Akt was not detected in U251N and U87MG cells (Data not shown). Further, the expression level of total c-Met was not reduced by shMet-expressing Ads in HDF normal cells (Supplementary Figure S2A). These results demonstrate that c-Met-specific shRNA-expressing Ads can significantly suppress the level of c-Met expression as well as downstream signaling of c-Met in cancer cells, and further suggest that dual shRNA expression system is more effective in suppressing the expression of c-Met than single shRNA expression system.

### c-Met-specific shRNA-expressing Ads inhibit cell proliferation and induce senescence-like phenotype

**Figure 3:**
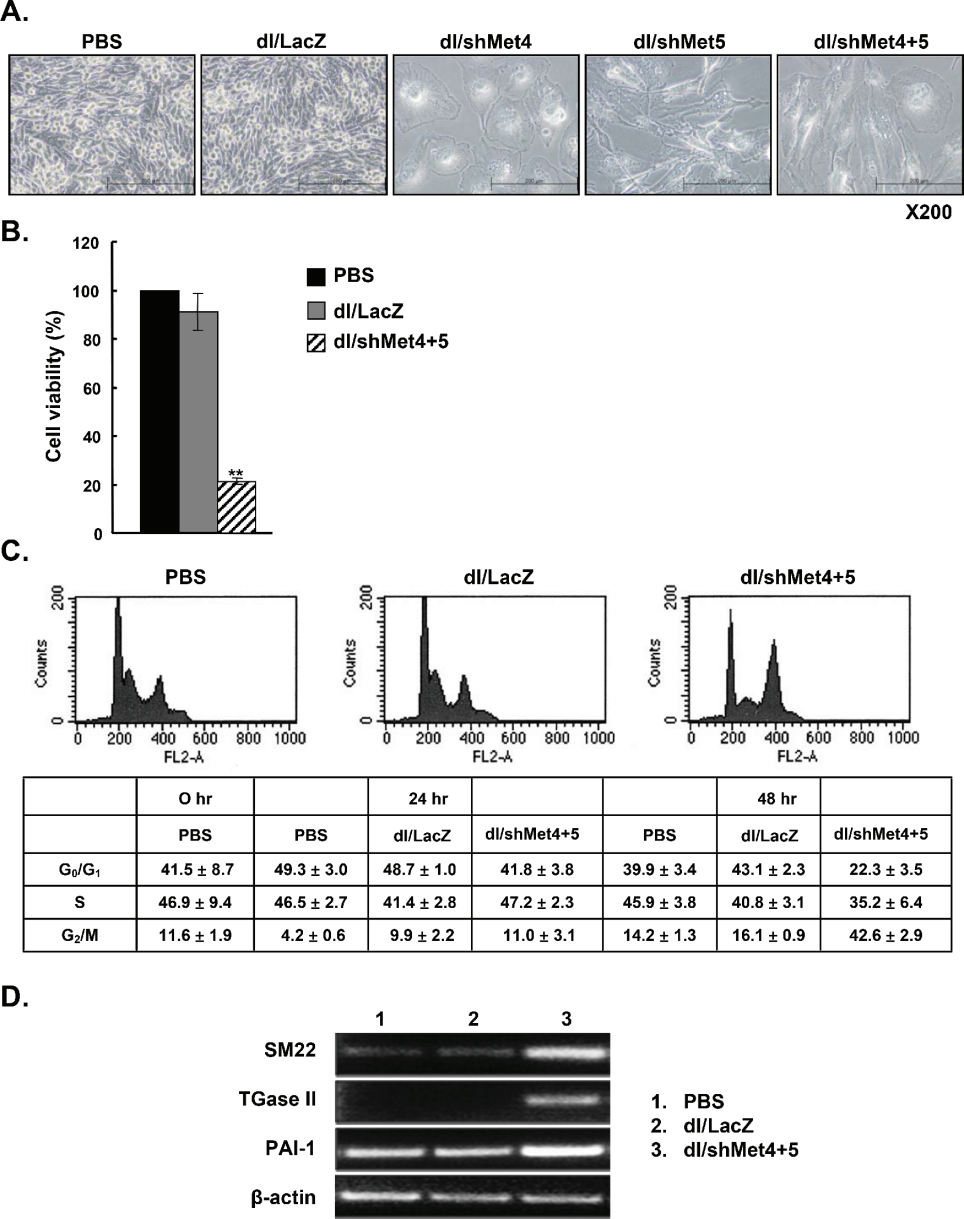
Induction of a senescence-like phenotype by c-Met-specific shRNA-expressing Ad **(A)** Light microscopic observation of U343 cells treated with dl/LacZ, dl/shMet4, dl/shMet5, or dl/shMet4+5. Original magnification: × 200. **(B)** Effect of c-Met-specific shRNA-expressing Ad on the viability of human glioma cells. U343 cells were transduced with dl/LacZ or dl/shMet4+5 at 100 MOI. After 3 days for treatment, cell viability was measured by MTT assay. Results are expressed as a percentage of control (PBS-treated cell) and values represent means ± SE. **P < 0.01, compared with PBS- or dl/LacZ-treated cells. **(C)** Changes in DNA contents following transduction with dl/LacZ or dl/shMet4+5 at an MOI of 30. Cells were fixed with ethanol and their DNA contents were measured by FACS analysis. Representative histograms of three independent experiments are shown. Percentages of G_0_/G_1_-, S-, and G_2_/M-phase cells were calculated by deconvolution of the DNA content histograms. Values are presented as the mean ± SE of three independent experiments. **(D)** RT-PCR analysis of gene products associated with cellular senescence. RNA was prepared from U343 cells transduced with dl/LacZ or dl/shMet4+5 at an MOI of 100, and the mRNA levels of senescence-related genes such as SM22, TGase II, and PAI-1 were detected.

To gain insights into functional changes of these newly engineered Ads, inhibition of cell proliferation using the MTT assay was first examined. First as shown in Figure 3A visually, dl/shMet4-, dl/shMet5-, or dl/shMet4+5-transduced cancer cells showed decreased cell confluence, and exhibited enlargement of cell volume (lamellafodia) and flattened shape. Consistently, significant reduction in cell viability (60–75%) (*P* < 0.01) was observed (Figure 3B). This decrease in cell viability by shMet was also observed in the other cancer cell lines tested (data not shown). In contrast with cancer cells, the cell viability of HDF normal cells was not changed after transduction with dl/shMet4+5 (Supplementary Figure S2B).

Next, to determine the mechanism of shMet-induced decrease in cell viability, induction of apoptotic cascade was assessed by TUNEL analysis. Interestingly, there was no difference in the percentage of TUNEL-positive cells after transduction with dl/shMet4+5 versus dl/LacZ (data not shown). In contrast, FACS analysis revealed that the population of cells in G_2_/M arrest dramatically increased to 42.6 ± 2.9% (dl/shMet4+5) as compared to dl/LacZ-transduced cells (16.1 ± 0.9%). Moreover, percentage of cells in G_0_/G_1_-phase decreased to 22.3 ± 3.5% (dl/shMet4+5) as compared with dl/LacZ-transduced cells (43.1 ± 2.3%) (Figure 3C). Cells in S-phase also showed slight reduction (5.6%).

To examine whether these phenomena were related to senescence, we examined the expression levels of gene products commonly associated with cellular senescence by RT-PCR. The mRNAs level of senescence-related genes such as SM22, TGase II, and PAI-1 were significantly increased in the cells transduced with dl/shMet4+5 compared with cells transduced with dl/LacZ (Figure 3D). Taken together, these results indicate that the major mechanism of cell death induced by knockdown of c-Met is not apoptosis, rather the induction of senescence.

### Induction of autophagy by shMet-expressing Ads

**Figure 4:**
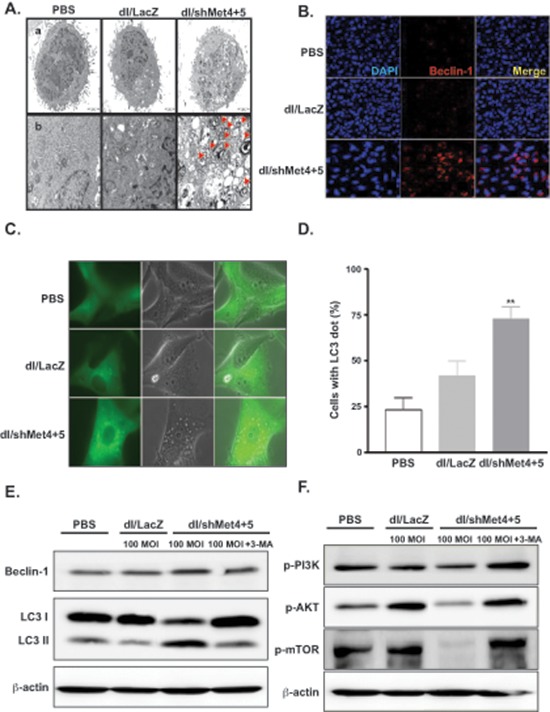
Induction of autophagy in glioma cells transduced with c-Met-specific shRNA-expressing Ad **(A)** Electron photomicrographs showing the ultrastructure of U343 cells transduced with dl/LacZ or dl/shMet4+5 at an MOI of 50 for 72 hr. The arrowheads indicate autophagic vacuoles. Scale bar = 2 μm. Original magnification: × 5000 (top) and × 10000 (bottom). **(B)** Microscope image of Beclin-1. U343 cells were transduced with dl/LacZ or dl/shMet4+5 at an MOI of 30 for 72 hr. Cells were then labeled with anti-Beclin-1 (red) or DAPI (blue), and examined by confocal microscopy. Original magnification: × 400. **(C)** U87MG cells stably transfected with GFP-LC3 were transduced with dl/LacZ or dl/shMet4+5 at an MOI of 100 for 48 hr. And then the translocation of GFP-LC3 was observed. Representative images of the cells were observed under fluorescence and phase-contrast microscopy. Original magnification: × 600. **(D)** The percentage of cells with GFP-LC3 localized to granular structures was estimated by counting a minimum of 100 cells per sample, and data is represented as means ± SE of five independent experiments. **P < 0.01 compared with PBS-treated and dl/LacZ-transduced cells. **(E)** Involvement of LC3 and Beclin-1 in shMet-expressing Ad-transduced glioma cells. U343 cells were pretreated with or without 3-MA (0.5 m*M*) for 30 min, and then transduced with dl/LacZ or dl/shMet4+5 at 100 MOI. Cells were then analyzed with antibody against Beclin-1 or LC3-I & -II (microtubule associated protein 1 light chain 3). **(F)** Suppression of c-Met-induced PI3K–AKT–mTOR signaling pathway. U343 cells were treated as indicated above (Figure 4E). After 48 hr, cell lysates were determined by western blot analysis with antibodies against p-PI3K, p-AKT, and p-mTOR.

Previous studies have shown that autophagy is activated during senescence, and its activation is correlates with negative feedback in the phosphatidylinositol 3-kinase (PI3K)–AKT–mTOR pathway [19]. In addition, a subset of autophagy-related genes is known to be up-regulated during senescence [20]. To examine the role of autophagy in cells transduced with shMet-expressing Ad, we analyzed the ultrastructure of U343 cells transduced with dl/shMet4+5 by TEM imaging. dl/shMet4+5-transduced cells showed abundance of vacuoles resembling autophagosomes without appearance of the chromatin condensation and the DNA fragmentation which are the characteristics of apoptosis (Figure 4A). Moreover, it was observed that cytoplasmic content and organelles in dl/shMet4+5-transduced cells are sequestered by double-membrane bound vesicles. Since autophagy is the process of sequestrating cytoplasmic proteins into the lytic component and is characterized by the formation of acidic vesicular organelles (AVOs) [21], dl/LacZ- or dl/shMet4+5-transduced cells were stained with acridine orange. As shown in Supplementary Table 2, dl/shMet4+5 at an MOI of 30, 50, and 100 increased the percentage of AVOs from 7.2% to 18.97%, 20.44%, and 22.88%, respectively (**P < 0.01 versus PBS, *P < 0.05 versus dl/LacZ group). In contrast, there was no statistical difference between PBS-treated and dl/LacZ-transduced tumor cells. Consistent with this observation, dl/shMet4+5 also caused accumulation of a key regulator of autophagy, Beclin-1, into intracytoplasmic structures (Figure 4B).

### Involvement of LC3 in shMet-expressing Ad-transduced glioma cells

LC3 (microtubule-associated protein 1 light chain 3, or shortly LC3) is involved in autophagic vesicle formation, thus it has been recently proposed to serve as a marker for autophagic vesicles and autophagic activity [22]. We thus analyzed the accumulation of LC3 in dl/LacZ- or dl/shMet4+5-transduced cells. In PBS- or dl/LacZ-treated cells, GFP-LC3 showed diffuse distribution of GFP expression, whereas ring-shaped pattern around the vacuoles and high-intensity punctate expression was observed in the shMet4+5-transduced cells at 24 hr (data not shown) and at 48 hr (Figure 4C and 4D).

During the process of autophagy, Beclin 1-PI3K complex is thought to undergo subcellular distribution to a membrane structure, which eventually leads to the recruitment of autophagy proteins and the proper conjugation of LC3 to membrane. In addition, the PI3 kinase complex (class III) including Beclin-1/Atg6 is required for the generation of preautophagosome structures [23]. We thus investigated the expression level of two key autophagy genes, LC3 and Beclin 1. Beclin-1 expression was increased when cells were transduced with dl/shMet4+5 compared with PBS- or dl/LacZ-treated cells (Figure 4E). In addition, the ratio of LC3-II to LC3-I in shMet4+5-transduced cells was dramatically increased by transduction with dl/shMet4+5, showing increased expression of LC3-II and decreased expression of LC3-I compared with PBS- or dl/LacZ-treated cells. Moreover, the increase of LC3-II gene expression and the decrease of LC3-I gene expression was blocked completely by the treatment with 3-MA, which is inhibitor of autophagosome formation [24]. However, transduction with control Ad, dl/LacZ did not change the ratio of LC3-I to LC3-II in either cell type compared with PBS treatment. These results, together with those of the electron microscopic analysis and acridine orange staining, indicate that shMet4+5-expressing Ad induces autophagy in malignant glioma cells.

### Suppression of c-Met-induced PI3K–AKT–mTOR signaling pathway

Recent evidence indicates that the phosphatidylinositol 3-kinase (PI3K)–AKT–mTOR pathway is closely linked with autophagy [25]. Therefore, we assessed the effect of shMet4+5-expressing Ad on the PI3K–AKT–mTOR signaling pathway by measuring the levels of downstream targets gene. As shown in Figure 4F, the expression levels of p-PI3K, p-AKT, and p-mTOR were notably reduced in U343 cells transduced with dl/shMet4+5. The decreased PI3K–AKT–mTOR pathway in dl/shMet4+5-transduced cells was reversed after treatment with 3-MA, indicating that shMet expression from Ad inhibits the PI3K–AKT–mTOR signaling pathway. Further, the expression level of phospho-S6, the ribosomal protein that is phosphorylated in response to mTOR activation, was also slightly reduced by p-mTOR inhibition in dl/shMet4+5-treated cells compared to dl/LacZ-infected control group.

### Enhanced anti-tumor effect of shMet-expressing Ads

**Figure 5:**
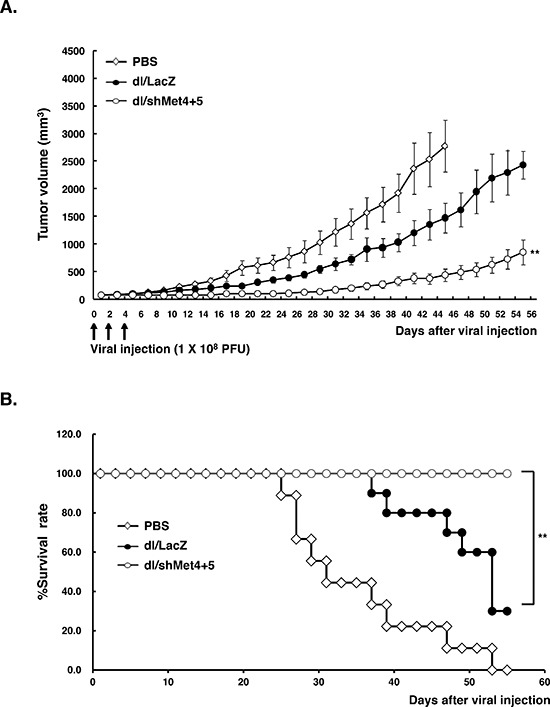
c-Met-specific shRNA-expressing Ad effectively inhibits tumor growth *in vivo* **(A)** Potent antitumor efficacy of c-Met-specific shRNA-expressing Ad. When U343 human glioma xenograft tumor size reached an average size of 70–100 mm^3^, tumors were injected with PBS (◊), dl/LacZ (•), or dl/shMet4+5 (○). Tumor growth was monitored and recorded every other day until the end of the study. The arrow represents Ad inoculation. Values represent the means ± SE (7 animals per group). **P < 0.01 compared with PBS- or dl/LacZ-treated groups. **(B)** Overall survival curve. The percentage of surviving mice was determined by monitoring tumor growth-related events over a period of 55 days. Tumor size over 1500 mm^3^ was regarded as death. **P < 0.01 compared with PBS- or dl/LacZ-treated groups.

To evaluate the therapeutic potential of shMet-expressing Ads *in vivo*, dl/shMet4+5 was subsequently assessed for their ability to suppress the growth of U343 human glioma xenograft model established in nude mice. As seen in Figure 5A, the growth of tumors treated with dl/shMet4+5 was substantially delayed compared with those of PBS- or dl/LacZ-treated tumors. At 45 days post-treatment, tumors in the PBS-treated group reached an average tumor volume of 2771.6 ± 467 mm^3^ and those treated with dl/LacZ reached 1468.1 ± 268 mm^3^. In marked contrast, tumor growth was dramatically suppressed in mice injected with dl/shMet4+5 (433.3 ± 108 mm^3^; *P* < 0.01, versus PBS or dl/LacZ group). Throughout the course of the study, no systemic toxicity, such as diarrhea, loss of weight, or cachexia was observed.

The survival of Ad-treated mice was evaluated over a period of 50 days. Ads expressing shMet-treated animals showed a longer, significant improvement in overall survival ompared with either PBS- or dl/LacZ-treated animals. In particular, all animals in the dl/shMet4+5 group were still viable compared to only 20% in the dl/LacZ-treated group at 55 days after treatment (*P* < 0.01) (Figure 5B).

### Sh-met-expressing Ad induced autophagy *in vivo*

**Figure 6:**
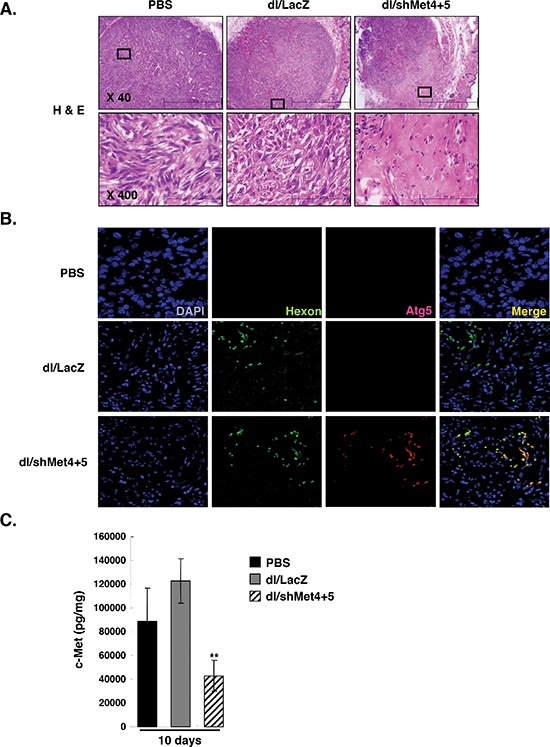
Histological and immunohistochemical examination of tumor tissues **(A)** Histological analysis of U343 tumors. At day 7 following three sequential treatments of dl/LacZ or dl/shMet4+5, mice were sacrificed and the sections of paraffin embedded tumor tissue from each group were stained with hematoxylin and eosin (H & E). Original magnification: × 40 and × 400. **(B)** Immunohistochemial staining for DAPI, Ad hexon, or Atg5. Original magnification: × 400. **(C)** Quantitative ELISA showing c-Met expression in tumor tissues treated with dl/LacZ or dl/shMet4+5. At day 7 following three sequential treatments of dl/LacZ or dl/shMet4+5, tumor tissues were homogenized and c-Met expression was quantified by conventional ELISA. Data represent the means ± SE of c-Met levels for seven mice per group. **P < 0.01 compared with PBS- or dl/LacZ-treated groups.

To verify the proposed mechanism of action of the c-Met-specific shRNA-expressing Ad, tumors were further investigated by histological examination. Tumors were harvested from each treatment group at day 7 following three sequential treatments. H&E staining revealed that most of the tumor mass remaining after treatment with dl/shMet4+5 were highly necrotic, whereas necrotic lesions were barely detectable in tumors treated with PBS or dl/LacZ (Figure 6A). Next to determine the mechanistic role of autophagy in tumor growth inhibition; immunohistochemical study was conducted using autophagy-specific Atg5, which is required for the elongation of autophagosomes. High level of Atg5 expression were observed in the tumor tissues treated with dl/shMet4+5, whereas the expression of Atg5 was very minimal in the tumor tissues treated with PBS or dl/LacZ (Figure 6B). Interestingly, expression of Atg5 co-localized with Ad hexon protein, providing additional proof that the region of the tumor tissues treated with dl/shMet4+5 represents autophagic cell death. Consistently, a marked suppression of c-Met expression was also observed in the tumor tissues treated with dl/shMet4+5 following 10 days of treatment, showing 56.4% reduction compared to dl/LacZ-treated control tumors (Figure 6C). Taken together, these results demonstrate that the potent antitumor efficacies of dl/shMet4+5-treated groups are susceptible to Ad-mediated cell death via autophaghy *in vivo*.

## DISCUSSION

c-Met and its ligand, HGF, are deregulated in many cancers and has been shown to be an important driver of primary tumor growth and metastasis (32). As such there has been great interest and number of therapeutic approaches such as small-molecule inhibitors, peptide antagonists, RNA-directed blockers, and monoclonal antibodies to abrogate c-Met and HGF signaling (33). Ad expressing shMet, specifically targeting c-Met, represents another innovative targeted therapeutic approach. Target recognition by siRNA was initially thought to be a highly sequence-specific as a single-nucleotide mismatch was able to abolish the gene silencing effect of the parental molecule. Thus, siRNAs may be used to suppress expression of point-mutated genes and provide a basis for cancer targeting therapy. To enhance target knock-down efficiency, we have engineered a dual silencing Ad expressing c-Met-specific shRNAs (Figure 1). The sequences for c-Met-specific shRNAs are located in immunoglobulin-plexin-transcription (IPT) repeat domain and juxtamembrane domain of c-Met, which is essential for induction of c-Met activation and the JM domain of RTKs has been shown to be a key regulator of kinase catalytic activities [4]. Thus, silencing of two major functional domains that regulate HGF/c-MET signaling represents a promising therapeutic strategy in targeting this driver of cancer.

Results from c-Met ELISA and protein analysis demonstrate that all three c-Met-specific shRNA-expressing Ads (dl/shMet4, dl/shMet5, and dl/shMet4+5) were highly effective in knocking down c-Met expression in multiple glioma cell lines (Figure 2). Comparatively, the dual Ad-expressing shRNA system which target both IPT repeat domain and juxtamembrane domain resulted in greater suppression compared to single targeting Ad-expressing shRNA. This is consistent with what has been reported recently from other investigators in which multiple shRNAs-combined vector systems were demonstrated to be much more efficient in silencing their gene of interest. For example, Ter Brake et al. showed that the multi-effective shRNAs elicited greater inhibition of HIV-1 production than a single shRNA, implying that such RNAi-based combinational approaches would result in a greater magnitude of inhibition along while decreasing possibilities of escape from the inhibition [26]. Taken together, these data provide strong support that multi-targeting shRNAs may be more efficient than a single shRNA expression system.

To date, much of the work in understanding the underlying mechanism of c-Met knockdown in cancer has predominantly focused on apoptosis-mediated cell death [7, 27]. Interestingly, we did not observe these apoptosis-mediated events following infection with shMet-expressing Ads, whether analyzed by flow cytometric analysis, TUNEL, or morphologic observation under microscopes, suggesting that effects seen in this report in malignant gliomas may be mediated by a differing mechanism than apoptosis. In fact, shMet-expressing Ad-transduced cells showed appearance of many vacuolated cells (Figure 4A), upregulated Beclin 1gene expression (Figure 4B), and increase of LC3-II to LC3-I ratio (Figure 4E) which are classic hallmarks of autophagic cell death (39). Thus it appears that in human glioma cell lines, autophagy not apoptosis may play a critical role in mediating cell death by shMet-expressing Ad treatment.

Autophagy is characterized by the accumulation of autophagic vacuoles, which is defined as the degradation of normal proteins in response to nutrient deprivation or other types of stresses (29, 38). It has also been identified as a mode of cell death during Ad infection and has been characterized as a mechanism of cell death or replication aid for other types of viruses (24). Consistent with what was observed *in vitro*, examination of xenograft tumors in mice also showed that Atg5 (autophagy regulator) was upregulated in shMet-expressing Ad-treated groups and co-localized with Ad proteins (Figure 6B).

Previously, targeting c-Met resulted in reversal of malignant phenotype, inhibition of tumor growth and metastasis (34, 35). In these studies, the proposed mechanism of tumor cell death was primarily attributed to the induction of apoptosis (34–36). Interestingly however, in the case of malignant gliomas, cells are resistant to caspase-mediated apoptosis, and our results point toward mTOR signaling pathway involved in mediating autophagy. Previous studies reported that autophagy is activated during the process of senescence, and these two pathways are functionally intertwined [19–20]. Senescence is a stable form of cell cycle arrest that enforces a potent barrier to tumorigenesis [28–32]. The activation of autophagy peaks in the transition phase of senescence and correlates with down-regulation of mTOR signaling pathway. In accordance with these previous observations, we observed that dl/shMet4+5 enhanced the expression levels of senescence-related genes such as SM22, TGase II, and PAI-1 (Figure 3D), demonstrating that the suppression of c-Met induces senescence.

As shown in Figure 4F, the level of phospho-mTOR (active form) was significantly decreased in response to treatment with shMet-expressing Ad, suggesting that c-Met repression contributed to the activation of autophagy by blocking auto-phosphorylation of mTOR. Although not conclusive, molecular mechanism linked to observed apoptotic resistance in this tumor type seems to involve the activation of the PI3K, Akt, and mTOR signaling pathway (37). Further, shMet-expressing Ad caused a significant inhibition of cell cycle progression (Figure 3C), resulting in a clear increase in the percentage of cells in the G2/M phase when compared with the non-transduced control cells. Similar responses were also observed in the previous studies showing that G2/M arrest was induced when cells were inhibited for PI3K/AKT/mTOR pathway [33, 34]. AKT promotes cell cycle progression at the G2/M transition through the suppression of the cyclin-dependent kinase Cdc2 and Cdc25C [35, 36]. Thus, dl/shMet4+5-mediated inhibition of AKT might induce the accumulation of inactive phospho-Cdc2 and phospho-Cdc25C, leading to subsequent G2/M arrest. Gerogenic (senescent) and quiescent cells can be distinguished by the level of phosphorylated S6, high in senescent cells and low in quiescent cells [37, 38] Although shMet-expressing Ad induced marked inhibition of auto-phosphorylation of mTOR, the expression level of phospho-S6 was not significantly attenuated (Supplementary Figure S3), indicating that cells become senescent when cell cycle is arrested.

Glioblastoma are highly invasive and rapidly spreading form of central nervous system tumor that are resistant to surgical and medical treatment. Several previous reports demonstrated an accelerated rate of spontaneous tumor development in murine models with disruption of autophagy-related genes, providing evidence that autophagy is a novel mechanism of cell-growth control and tumor suppression [39–41]. Of particular interest are recent findings that high-grade gliomas have lower expression of Beclin 1 when compared with low-grade gliomas [42]. The importance of autophagy in glioblastoma is also implicated by recent reports on another gene, *LC3*, considered to be the most reliable marker of autophagy. High *LC3* expression was shown to correlate with improved survival in glioblastoma patients with poor performance score [43]. Standard treatment for high-grade gliomas patients, i.e., maximal surgical removal of the tumor and postoperative radiation therapy (RT) combined with temozolomide chemotherapy (TMZ CHT) only provides marginal benefit. This lack of effect seems to be linked to high resistance of infiltrative tumor cells to apoptosis. As a result, there has been much interest in identifying therapeutics that can enhance apoptosis as well as discovering novel targetable approaches to induce cell death via non-apoptotic cell death. Recent studies indicate that high-grade gliomas cells are more sensitive to several treatments that induce autophagy. Thus, development of autophagy-inducing treatments (as proposed in this report) could be timely in improving the standard of care for malignant gliomas.

## MATERIALS AND METHODS

### Cell lines and cell culture

Human glioblastoma cell lines (U251, U343, and U87MG) and human dermal fibroblast (HDF) were purchased from the American Type Culture Collection (ATCC, Manassas, VA). All cell lines were grown in Dulbecco's modified high-glucose Eagle's medium (DMEM), supplemented with 10% fetal bovine serum (FBS), 2 m*M* L-glutamine, 1 m*M* sodium pyruvate, 1% MEM nonessential amino acids, penicillin-streptomycin (100 IU/ml), and Hank's balanced salt solution (HBSS). The cultures were incubated at 37°C in a humidified atmosphere of 5% CO_2_.

### Preparation and transfection of siRNAs specific to c-Met

Five siRNAs targeting human c-Met (GenBank accession number gi: 4557746) were designed using RNAi software (Ambion: www.ambion.com/techlib/misc/siRNA_finder.html) and synthesized by the Ambion Silencer TM siRNA construction kit (Cat #1620; Supplementary Table 1). To determine the most effective siRNA to knock down c-Met expression, 1 μg of the five synthesized siRNAs and a control siRNA specific to Lamin A/C was transfected into U343 cells (3 × 10^5^) in 6-well dishes using lipofectamine plus reagent (Invitrogen, Carlsbad, CA). Total cellular RNA was harvested at 48 hr after transfection, and analyzed for c-Met mRNA by semi-quantitative reverse transcription (RT)-polymerase chain reaction (PCR).

### Construction of Ads expressing c-Met-specific shrna

The siRNA-expressing vector pSilencer 2.1-U6 was purchased from Ambion (Ambion, Austin, TX) and used to construct Ads expressing human c-Met-specific shRNA. DNA oligonucleotides (Figure 1A) were specifically designed to generate the plasmid vector using the pSilencer system. To create c-Met shRNAs with 21-mer stems, sic-Met-4 (targeting position 1987–2007) or sic-Met-5 (3142–3162) was annealed to create a double-stranded oligonucleotides and was inserted into the pSilencer 2.1-hygro-U6 vector [17], resulting in pshMet4 and pshMet5, respectively. The U6-shMet4 and U6-shMet5 gene expression cassettes were then subcloned into the pSP72-E3 Ad shuttle vector [44], generating pSP72-E3/shMet4 and pSP72-E3/shMet5, respectively. To generate Ad expressing both shMet4 and shMet5, U6-shMet5 region was amplified by PCR with the following primer set: 5′-GTCAAGCTTGAATTCCCCAGTGGAAAGACG-3′ as the sense primer and 5′-GTCGAATTCAAGCTTCC AAAAAAAATTAGTTCG-3′ as the antisense primer. The PCR product containing U6-shMet5 was digested with *Hind*III, and then was subcloned into the pSP72-E3/shMet4, generating pSP72-E3/shMet4+5. The newly constructed pSP72-E3/shMet4, pSP72-E3/shMet5, and pSP72-E3/shMet4+5 Ad E3 shuttle vectors were linearized with *Xmn*I and co-transformed with a replication-incompetent Ad total vector (pdl/LacZ) into *Escherichia coli* BJ5183 for homologous recombination, generating the pdl/shMet4, pdl/shMet5, and pdl/shMet4+5 Ad vectors. Generation and purification of recombinant Ads expressing c-Met-specific shRNA were carried out as described previously [45].

### Enzyme-linked immunoadsorbent assay (ELISA)

The level of human c-Met was assessed using the Quantikine ELISA kits (c-Met: Biosource International Inc., Camarillo, CA) following the manufacturer's instructions. To generate conditioned media (CM), triplicate dishes of cells were transduced with phosphate-buffered saline (PBS), dl/LacZ, dl/shMet4, dl/shMet5, or dl/shMet4+5 at different MOIs for 48 hr. The media was then replaced with fresh media and cells were allowed to grow for another 24 hr. The CM from cells transduced with Ads was collected, and total cell protein was extracted and stored at –80°C for ELISA analysis. For the quantitation of c-Met in tumor tissues, tumor tissues were removed from mice treated with PBS, dl/LacZ, or dl/shMet4+5, and snap frozen in liquid nitrogen. Tissues were homogenized in ice-cold PBS with protein inhibitor cocktail (Sigma, St. Louis, MO), and analyzed for total protein content using a BCA protein assay reagent kit (Bio-Rad, Hercules, CA). The concentration of c-Met from CM and tissues were calculated from a standard curve derived using recombinant proteins.

### Cell proliferation assay

Inhibition of cancer and normal cell growth caused by c-Met shRNA was determined using MTT cell viability assay. MTT assay was performed as described previously [45]. Cells in 24-well plates were transduced with dl/shMet4, dl/shMet5, or dl/shMet4+5 at 100 MOI. At 72 hr post transduction, 250 μl of 3-(4, 5-dimethylthiazol-2-yl)-2,5-diphenyl-tetrazolium bromide (MTT; Sigma) in PBS was added to each well. After 4 hr incubation at 37°C, the precipitate was dissolved with 1 mL of dimethylsulfoxide (DMSO). The color intensity was then read on a microplate reader at 540 nm. All assays were performed in triplicate.

### Cell cycle analysis

Cells were transduced with dl/LacZ or dl/shMet4+5 at an MOI of 30. At 24 or 48 hr after transduction, floating cells were pooled, washed with PBS, and fixed in 70% (v/v) ethanol. Then, 0.5 ml of propidium iodide (PI) solution (50 mg/ml) was added and cells were left on ice for 15 to 30 min. DNA content was analyzed with FACS LSRII (BD Biosciences, San Jose, CA) as previously described [46], and data were analyzed using FACSDiva software.

### Reverse transcription (RT)-PCR

RT-PCR was used to determine the mRNA level of three genes that undergo senescence-related genes such as SM22, TGase II, and PAI-1. RT-PCR was performed as described previously [47]. PCR primers are described in Table 1.

### Electron microscope (EM) cytology

To morphologically demonstrate the induction of autophagy in c-Met shRNA-expressing Ad-treated tumor cells, we performed the ultrastructural analysis as described previously [48]. U343 cells were transduced with dl/LacZ or dl/shMet4+5 at an MOI of 50. Three days post-transduction, cells were centrifuged gently (3,000 rpm for 5 min) to form a pellet. After washing with PBS, pellets were fixed in 2.5% glutaraldehyde in 0.1 M sodium cacodylate buffer (pH 7.3) for 4 hr at 4°C. After washing in PBS, the cells were post-fixed in OsO4 and embedded in Epon812, and examined using an electron microscope (EM 902A, Zeiss, Oberkochen, Germany).

### Detection of acidic vesicular organelles (AVOs) with acridine orange

To detect and quantify AVOs in c-Met shRNA-expressing Ad-transduced cells, we performed vital staining using acridine orange as previously described [22]. The intensity of the red fluorescence is proportional to the degree of acidity and/or the volume of the cellular acidic compartment. U343 glioma cells in 6-well plates were treated with 30, 50, and 100 MOI of dl/LacZ or dl/shMet4+5. After 72 hr, cells were stained with acridine orange at a final concentration of 1 mg/mL for a period of 15 min, and analyzed by BD LSRII flow cytometry (BD Biosciences).

### Immunofluorescence assay

For immunofluorescence, cultured cells were first fixed and permeabilized. They were then blocked and incubated with Beclin 1. Staining was visualized by Alexa Flour 546-conjugated goat anti-rabbit IgG secondary antibody under a confocal laser-scanning microscope (LSM510, Carl Zeiss MicroImaging, Thornwood, NY). Nuclei were counterstained and visualized using DAPI stain (1 μg/ml, Sigma).

### Translocation of GFP-LC3 to autophagosomal structures

U87MG cells were transfected with GFP-LC3 encoding plasmid (21), and stable clones were selected using aminoglycoside antibiotic, G418 (500 μg/mL, Gibco, Grand Island, NY) [49]. To detect the localization of LC3, stable GFP-LC3-expressing cells were treated with 100 MOI of dl/LacZ or dl/shMet4+5 for 48 hr. Translocation of GFP-LC3 from cytosol to autophagic vacuoles was then observed under a fluorescence microscope using Zeiss filter set #10 (excitation band pass, 450–490 nm; emission band pass, 515–565 nm).

### Immunoblotting analysis for signaling molecules

Human glioma cells (U343, U251N, and U87MG) and HDF grown in 100-mm plate were transduced with dl/LacZ, dl/shMet4, dl/shMet5, or dl/shMet4+5 (200 MOI for U251N, U87MG, and HDF, 100 MOI for U343) in DMEM with 1% fetal bovine serum for 24 to 48 hr. Immunoblotting were performed as described previously [18]. Blocked membranes were incubated with antibodies against total c-Met, phospho-c-Met (Tyr1234/1235), Beclin I, LC3-I/II, p-Erk1/2 (Thr202/Tyr204), p-PI3K (Tyr458/Tyr199), p-Akt (Ser473), or p-mTOR (Ser2448) (each diluted 1:000, Cell Signaling Technology) overnight at 4°C. The secondary antibodies, goat anti-rabbit IgG horseradish peroxidase (HRP), goat anti-mouse IgG HRP, or mouse anti-goat IgG HRP (Cell Signaling Technology) were added for 60 min at room temperature. Finally, the blots were developed using enhanced chemiluminescence (ECL) (Amersham Pharmacia Biotech, AB, Uppsala, Sweden).

### Anti-tumor efficacy in human xenograft model

Male athymic nu/nu mice (5–6 weeks of age) were obtained from Charles River (Yokohama, Japan). All mice were housed and handled in accordance with the Animal Research Committee's Guidelines at Yonsei University College of Medicine, and all facilities are approved by AAALAC (association of assessment and accreditation of laboratory animal care). Mice were implanted subcutaneously with 2 × 10^7^ U343 human glioma cells. When tumors reached an average size of 70 – 100 mm^3^, Ads were administered intratumorally (1 × 10^8^ PFU) on days 1, 3, and 5. Tumor volume was calculated using the following formula: volume = 0.523*LW^2^*.

### Evaluation of tumor xenograft by histology and immunohistochemistry

Tumor tissues were fixed in 4% paraformaldehyde and embedded in paraffin for histological and immunohistochemical staining. Representative sections were stained with hematoxylin and eosin, and examined by light microscopy. Immunohistochemistry were performed as described previously [18]. The tumor sections were double immunostained with goat anti-Ad hexon (Chemicon International, Temecula, CA) and rabbit monoclonal anti-Atg5 (Epitomics, Burlingame, CA).

### Statistical analysis

The data are expressed as mean ± SE, and the significance of differences between group means was determined by the Mann-Whitney test (nonparametric rank sum test) using Stat View software (Abacus Concepts, Inc., Berkeley, CA). Differences were considered significant when *P* < 0.05.

## SUPPLEMENTARY FIGURES AND TABLES



## Abbreviations

RTK: receptor tyrosine kinase
HGF: hepatocyte growth factor
LC3: microtubule-associated protein1 light chain 3
RNAi: RNA interference
shRNA: short hairpin RNA
Ad: adenovirus
IPT: immunoglobulin-plexin-transcription
RT: radiation therapy
