# Supplementary material for: Adenovirus expressing dual c-Met-specific shRNA exhibits potent antitumor effect through autophagic cell death accompanied by senescence-like phenotypes in glioblastoma cells
Source: Oncotarget. 2015 Feb 17;6(6):4051–65. doi: 10.18632/oncotarget.3018 (PMC4414172; doi:10.18632/oncotarget.3018)
Supplement: Supplementary file 1 [file oncotarget-06-4051-s001.pdf]

SUPPLEMENTARY FIGURES AND TABLES

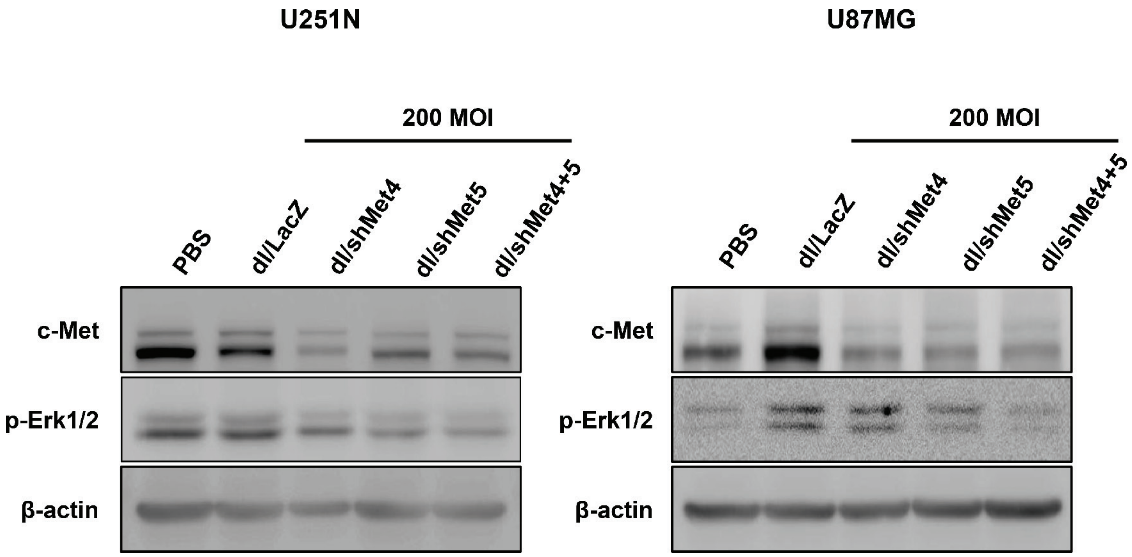

Supplementary Figure S1: The expression of total c-Met and phospho-Erk1/2 in glioma cells transduced with c-Met-specific shRNA-expressing Ad. Human glioma cancer cells (U251N and U87MG) were transduced with dl/LacZ, dl/shMet4, dl/shMet5, or dl/shMet4+5 at 200 MOI. After 72 hr, cell lysates were assessed for total c-Met and phospho-Erk1/2 by western blot analysis. Results are representative of three independent experiments.

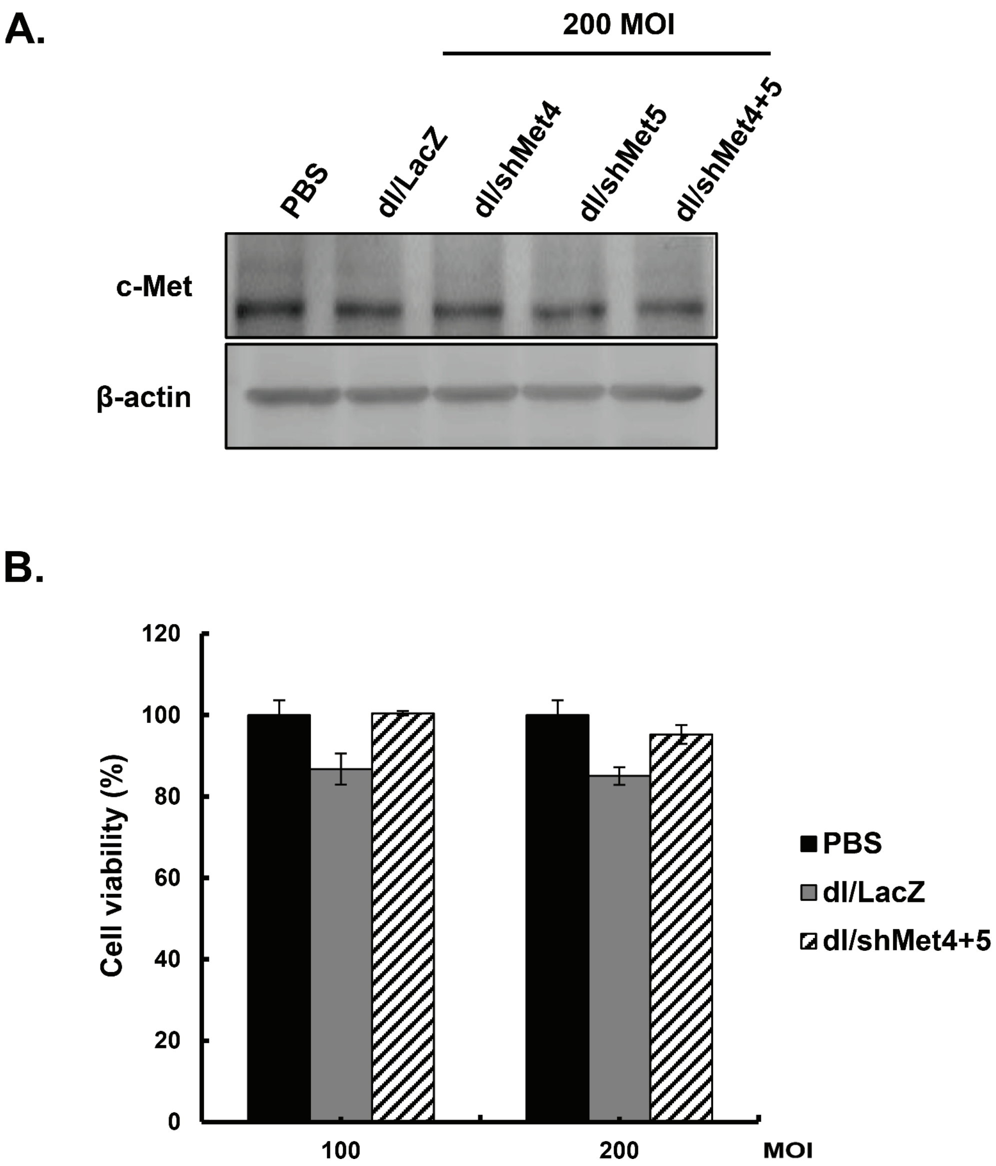

Supplementary Figure S2: The c-Met expression and viability of normal cell transduced with c-Met-specific shRNA-expressing Ads. (A) HDF cells were transduced with dl/LacZ, dl/shMet4, dl/shMet5, or dl/shMet4+5 at 200 MOI. After 72 hr, total c-Met and phospho-c-Met expression level was observed by western blot analysis. Results are representative of three independent experiments. **(B)** Effect of c-Met-specific shRNA-expressing Ad on the viability of human normal cells. HDF normal cells were transduced with dl/LacZ or dl/shMet4+5 at 100 or 200 MOI for 72 hr. Result are expressed as a percentage of control (PBS-treated cells).

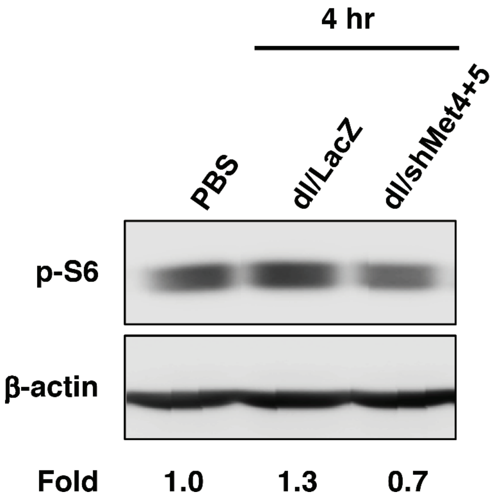

Supplementary Figure S3: The expression level of phospho-S6. U343 cells were transduced with dl/LacZ or dl/shMet4+5 at 100 MOI. After 4 hr, cells were analyzed with antibody against phospho-S6. Results are representative of three independent experiments.

Supplementary Table 1: Primer information for cellular senescence gene

Gene	Primer sequence	Product (bp)
**SM22**		
Forward	5′-TGGCGTGATTCTGAGCAA-3′	534
Reverse	5′-CTGCCAAGCTGCCCAAGG-3′	
**TGase II**		
Forward	5′-CTCGTGGAGCCAGTTATCAACAGCTAC-3′	310
Reverse	5′-TCTCGAAGTTCACCACCAGCTTGTG-3′	
**PAI-1**		
Forward	5′-GTGTTTCAGCAGGTGGCGC-3′	300
Reverse	5′-CCGGAACAGCCTGAAGAAGTG-3′	
**β-actin**		
Forward	5′-CGTCTTCACCATGGAGA-3′	310
Reverse	5′-CGGCCATCACGCCCACAGTTT-3′	

Supplementary Table 2: Development of AVOs in PBS-, dl/lacZ-, or dl/shMet4+5-treated U343 cells. Tumor cells were transduced with dl/lacZ or dl/shMet4+5 at an MOI of 30, 50, and 100 for 72 hr. Red fluorescence in acridine orange-stained cells was detected by FACS analysis.

	PBS	dl/LacZ	dl/shMet4+5
30 MOI	7.22 ± 1.19	11.28 ± 0.71	18.97 ± 1.75
50 MOI	7.22 ± 1.19	11.08± 0.1	20.44 ± 0.62
100 MOI	7.22 ± 1.19	14.47 ± 3.26	22.88 ± 2.29
